# A Review on the Design of Carbon-Based Nanomaterials as MRI Contrast Agents

**DOI:** 10.3390/molecules29071639

**Published:** 2024-04-05

**Authors:** Sarah Garifo, Thomas Vangijzegem, Dimitri Stanicki, Sophie Laurent

**Affiliations:** 1 NMR and Molecular Imaging Laboratory, General, Organic and Biomedical Chemistry Unit, University of Mons, 19 Avenue Maistriau, 7000 Mons, Belgium; thomas.vangijzegem@umons.ac.be (T.V.); dimitri.stanicki@umons.ac.be (D.S.); 2 Center for Microscopy and Molecular Imaging (CMMI), 8 Rue Adrienne Boland, 6041 Gosselies, Belgium

**Keywords:** magnetic resonance imaging, contrast agents, carbon-based nanomaterials, nanodiamonds, nanotubes, fullerene, graphene

## Abstract

The administration of magnetic resonance imaging (MRI) contrast agents (CAs) has been conducted since 1988 by clinicians to enhance the clarity and interpretability of MR images. CAs based on gadolinium chelates are the clinical standard used worldwide for the diagnosis of various pathologies, such as the detection of brain lesions, the visualization of blood vessels, and the assessment of soft tissue disorders. However, due to ongoing concerns associated with the safety of gadolinium-based contrast agents, considerable efforts have been directed towards developing contrast agents with better relaxivities, reduced toxicity, and eventually combined therapeutic modalities. In this context, grafting (or encapsulating) paramagnetic metals or chelates onto (within) carbon-based nanoparticles is a straightforward approach enabling the production of contrast agents with high relaxivities while providing extensive tuneability regarding the functionalization of the nanoparticles. Here, we provide an overview of the parameters defining the efficacy of lanthanide-based contrast agents and the subsequent developments in the field of nanoparticular-based contrast agents incorporating paramagnetic species.

## 1. Introduction

Magnetic resonance imaging (MRI) is a powerful non-invasive bio-imaging technique used daily in the medical field due to its high spatial resolution. MRI is essentially based on water protons’ nuclear magnetic resonance, due to its high abundance in the body. However, the natural contrast is not always sufficient to allow for efficient differentiation between different tissues on MR images, so contrast agents (CAs) must be used. Those contrast agents will decrease the water proton relaxation times and can be divided in two categories: paramagnetic, T_1_, or “positive CAs” and superparamagnetic, T_2_, or “negative CAs” [1,2]. T_2_ agents, mainly based on superparamagnetic iron oxide nanoparticles (SPIONs), preferentially decrease the T_2_ relaxation time of the water protons, inducing darkening the area of accumulation on the images. For this reason, they are, nowadays, no longer used by radiologists, but are still at the forefront of many research studies among the MRI community.

**Figure 1:**
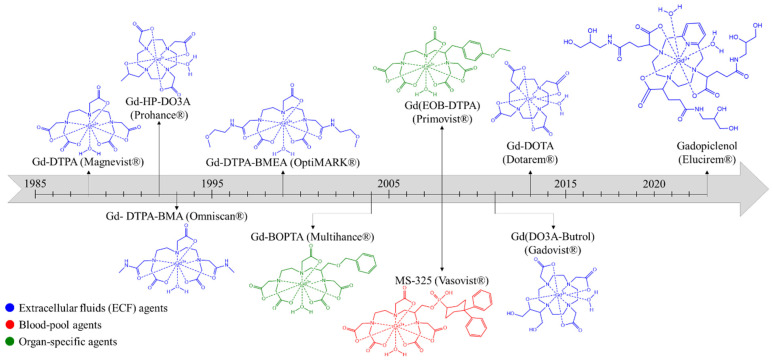
Chronological chart of clinical GBCAs and their FDA approval years (in the U.S.).

The first T_1_ contrast agent used and approved in MRI was based on a gadolinium complex. Indeed, gadolinium ions Gd^3+^ were chosen thanks to their paramagnetic state (*S* = 7/2 spin), and hence, their high capacity to modify water proton relaxation times. The first gadolinium complex developed and commercialized was the Gd-DTPA (Magnevist^®^). Nevertheless, at the end of the 20th century, Cowper et al. published and evidenced a relation between multiple injections of gadolinium-based contrast agents (GBCAs) and nephrogenic systemic fibrosis (NSF) in patients suffering from chronic kidney disease [2,3,4,5]. To respond to that issue, Gd-HP-DO3A based on gadolinium(III) *N*-(hydroxyethyl)-1,4,7-tris(carboxymethyl)-1,4,7,10-tetraazacyclododecane (Prohance^®^) was formulated, and the last developed and most used T_1_ contrast agent in the clinic is Gd-DOTA (Dotarem^®^), based on a macrocyclic 1,4,7,10-Tetraazacyclododecane-1,4,7,10-tetraacetate gadolinium complex [6]. Research is continually in development to increase the efficacy of GBCAs and, subsequently, to lower their required dose. Recently, the FDA approved a Gd complex based on the PCTA structure (Gadopiclenol (Elucirem^®^)) that was developed in this context. This structure allows the presence of two water molecules in the inner sphere, which, consequently, increases the relaxivity [7]. Nevertheless, it is important to keep in mind that recent studies have evidenced the presence in the brain of gadolinium species with a higher rate of accumulation in the case of linear complexes instead of macrocyclic ones, even in patients with no kidney disease [6]. In view of Gd complexes’ issues, researchers are continually developing alternatives with an equal or higher efficiency (Figure 1).

Other than using GBCAs, manganese-based MRI contrast agents are increasingly emerging as potentially safer alternatives for manganese-enhanced MRIs (MEMRI) [8,9,10]. Molecular Mn-based contrast agents behave similarly to Gd complexes, i.e., their safe use in the clinic is enabled by their complexation with chelating ligands. Hence, the development of innovative Mn-based contrast agents follows a similar pattern to that of GBCAs.

## 2. Paramagnetic Relaxation Theory

Paramagnetic complexes such as gadolinium complexes are mainly exploited for their doping effect on the longitudinal relaxation rate (R_1_) of water protons. Longitudinal relaxation time (T_1_) is defined as the time needed for the longitudinal magnetization M_Z_ vector to reach $1-e^{-t/T_{1}}\sim$ 2/3 of its equilibrium value M_0_ after a 90° pulse. T_1_ is measured by the inversion recovery sequence, which consists of an impulsion of 180°, followed by a delay t, a second impulsion at 90°, and finally, the recording of the free induction decay (FID). The longitudinal relaxivity, which is the slope of the decay rate R_1_ (inverse of T_1_) versus the metal (M) molar concentration gives access to the efficiency of the complexes (Equation (1)).

(1) $$r_{1}=\frac{\left(\frac{1}{T_{1}}\right)-R_{1(water)}^{d}}{\left[M_{complex}\right]}\;s^{-1}\cdot {mM}^{-1}$$

where $R_{1(water)}^{d}$ represents the diamagnetic contribution to the decay rate of pure water.

In other words, relaxivity highlights the capacity of one millimole per liter of the contrast agent to decrease the relaxation time of water molecules present in its vicinity. The higher the relaxivity, the more efficient the contrast agent is in modifying the contrast of MR images. In the case of T_1_ agents like Gd complexes, a high relaxivity will be translated to images by an enlightenment of the area of accumulation, thanks to appropriate radiofrequency spin echo sequences with very short echo times, which operate a “T_1_-weighting” of the MR images.

Relaxivity depends on three mechanisms: (i) the inner sphere, which corresponds to the first coordination sphere of the metal containing one or several coordinated water molecule(s), (ii) the outer sphere, corresponding to the bulk water diffusing in the close vicinity of the complex, and (iii) the second sphere, coming from water molecules slightly bound to the complex through hydrogen bonds, for example. Since the outer sphere’s mechanism mainly depends on the diffusion of water, it cannot be easily tuned to increase the relaxivity, as only a temperature changes could significantly modify this diffusion. Concerning the inner sphere’s mechanism, it is of quantum mechanics origin and is governed by both scalar and dipolar interactions between the nuclear spins of water molecules and the orbital spins of the f or d-layer electrons of the paramagnetic center. The scalar interaction is negligible for all the Gd complexes and for most of the Mn-complexes [8], so only the dipolar contribution will determine the inner sphere’s mechanism. It is given by Equation (2), where *q* is the number of coordinated water molecule(s), τ_M_ is the residence time of these coordinated water molecules, and T_1m_ is the longitudinal relaxation time of the coordinated water molecule, as defined by Equation (3), according to the paramagnetic relaxation theory of Solomon–Bloembergen–Morgan.

(2) $$r_{1}^{\mathrm{is}}=\frac{q/[H_{2}O]}{T_{1m}+\tau_{M}}$$

(3) $$\frac{1}{T_{1m}}=\frac{2}{15}\left(\frac{\mu_{0}}{4\pi }\right)\frac{\gamma_{H}^{2}g_{e}^{2}\mu_{B}^{2}S(S+1)}{r_{\mathrm{MH}}^{6}}\left[\frac{7\tau_{c2}}{1+\omega_{S}^{2}\tau_{c2}^{2}}+\frac{3\tau_{c1}}{1+\omega_{H}^{2}\tau_{c1}^{2}}\right]$$

(4) $$Where\;\frac{1}{\tau_{c1,2}}=\frac{1}{\tau_{R}}+\frac{1}{\tau_{M}}+\frac{1}{\tau_{s1,2}}$$

where μ_0_ is the vacuum permeability; r_MH_ is the distance between the metal ion and the water hydrogen of the coordinated water molecule; γ_H_ is the proton magnetogyric ratio; g_e_ is the electronic gyromagnetic factor; μ_B_ is the Bohr magneton; ω_S,H_ is the Larmor frequency of electrons or protons; τ_s1,2_ are the longitudinal and transverse relaxation times; τ_c1,2_ is the correlation time for magnetic fluctuation; τ_R_ is the rotational correlation time; and τ_M_ is the water residence time in the inner coordination sphere.

**Figure 2:**
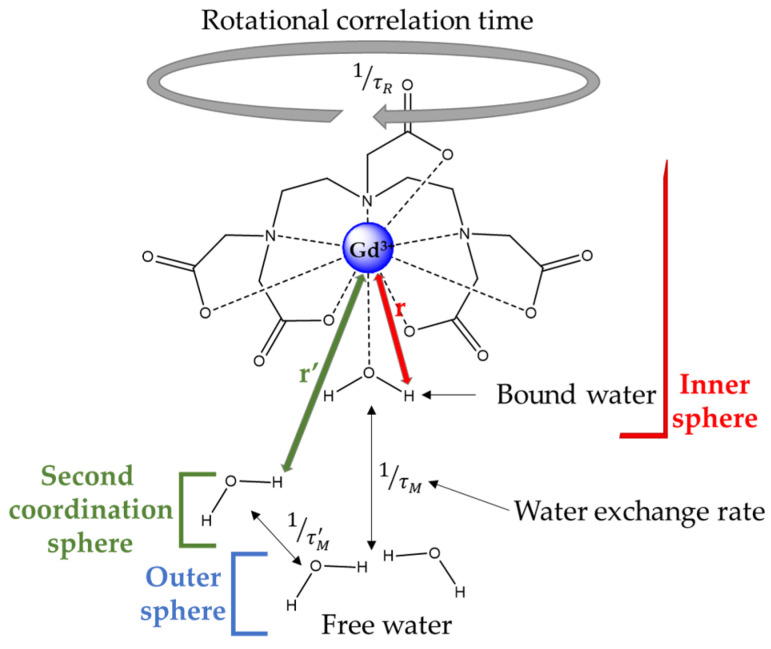
Schematic representation of mechanisms contributing to the relaxation efficacy.

Among all these parameters, three were proven to have a huge influence on the relaxivity at the magnetic fields used in the clinic (the clinical window): the number of coordinated water molecules (q), the rotational correlation time (τ_R_), and the water residence time in the inner sphere (τ_M_) (Figure 2). 

**Figure 3:**
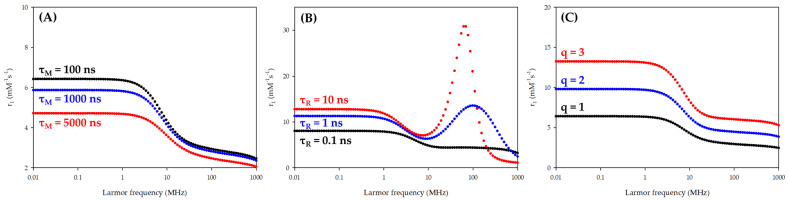
Influence of (**A**) τ_M_; (**B**) τ_R_; and (**C**) *q* on NMRD profiles of small Gd complexes.

In order to increase *q*, it is necessary to modify the ligand structure to decrease the number of donor sites and, hence, its denticity. Such modifications can, thus, lead to a decrease in the complex stability constant, which can result in its destabilization. On the other hand, t_R_ can be modified by grafting the small complexes on a bigger structure. Indeed, the larger the molecule, the longer its rotational correlation time will be, with a beneficial effect on the interaction between the nuclear and electronic spins, and, thus, on the relaxivity in the clinical window. Concerning τ_M_, the chemical nature of the donor groups of the ligand will have a huge influence. It has notably been shown that amide donor groups will lead to a longer water residence time in the inner sphere as compared to carboxylate groups, which has a detrimental effect on the relaxivity of the complex (as shown in Figure 3).

It has been recognized that the relaxivity of paramagnetic lanthanide-based contrast agents can be improved by optimizing the inner-sphere relaxivity mechanism in slowing down the tumbling of lanthanide chelates to influence the rotational correlation time (τ_R_). The challenge is closely correlated with the ability to generate a maximum effect on the complex motion of clinically approved compounds. Thus, a conventional strategy consists of increasing the size and the molecular weight of the ligand coordinating the lanthanide ion by applying structure modification or by entrapping agents. In recent years, research efforts have intensified on the engineering of sophisticated carriers that could carry conventional contrast agents. These structures include nanoscale dendrimers, liposomes, micelles, protein-based vectors, carbon nanosystems, and (in)organic nanoparticles. These strategies involve the confinement of paramagnetic complexes inside a matrix (nanoparticles) or linking to organic or inorganic surface structures.

Nanoparticles (NPs) are being increasingly studied because they provide versatile platforms for medical applications due to intrinsic properties such as a high surface area and biocompatibility, etc. Nanoparticles functionalized with paramagnetic species have been intensively investigated as MRI T_1_ contrast agents. Immobilization by the covalent grafting of paramagnetic complexes onto the nanoparticle surface allows for efficiently increasing the longitudinal relaxation process, resulting in an enhancement of the T_1_-weighted signal on MR images. A very broad range of nanoparticular MRI systems have been reported in the literature. Although several reviews have summarized the use of materials such as silica [11,12,13], polymers [14,15], or inorganic [16,17] nanoplatforms like CAs for MRI, the use of carbon-based nano-systems has been sparsely discussed. Hence, in this review, we discuss recent applications regarding the use of carbon-based nano-systems (such as carbon nanotubes, nanoparticles of diamonds, and fullerenes) as platforms for the loading or encapsulation of paramagnetic species and their subsequent use as CAs for MRI. Table 1 summarizes the studied nanosystems’ properties according to their starting material, their longitudinal relaxation value, measured conditions, and relative % of enhancement, starting from small-sized chelates.

## 3. Paramagnetic Carbon-Based Nanomaterials

**Figure 4:**
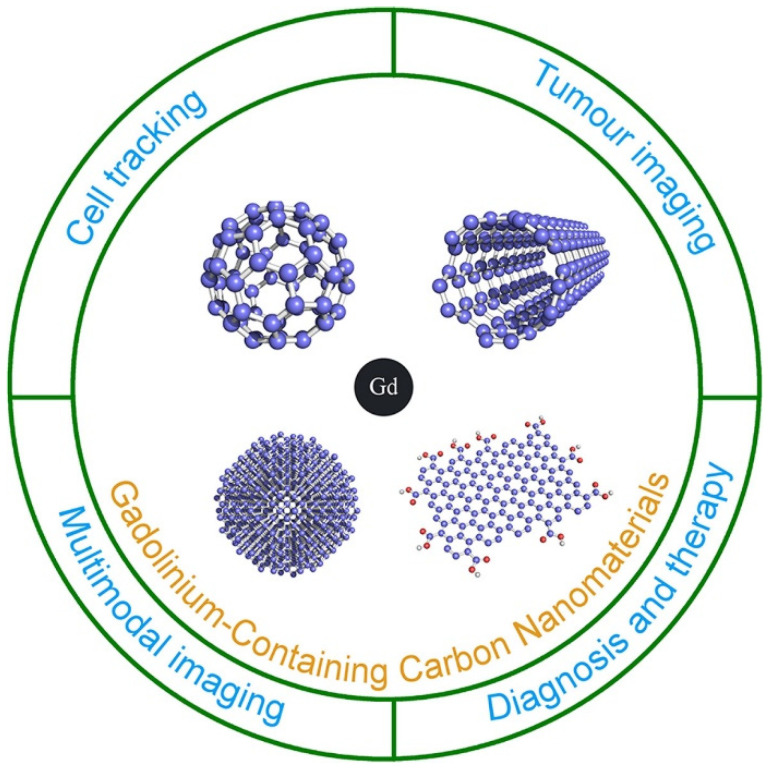
Illustration of the major biological applications of gadolinium-containing carbon nanomaterials (Reprinted with permission from [18]).

Recent advances in nanotechnology have attracted significant attention to carbon-based nanomaterials (CNMs) for MRI. CNMs can be chelated directly with gadolinium ions, for instance, to design a so-called gadonanomaterial. Different allotropic forms of carbon can be used to form efficient paramagnetic-based carbon nanomaterials, such as gadonanodiamonds, gadonanotubes, gadofullerenes, or gadographenes (Figure 4).

There have been numerous reports on paramagnetic CNMs over the past decade, and some have ranked among the greatest potent T_1_-proton relaxation agents for clinical field MRI, as reported herein. These promising CNMs achieve high relaxivities of up to 80 s^−1^ mM^−1^ normalized per paramagnetic centers, approaching or even exceeding some theoretical limits for a single bound water molecule (Table 1).

### 3.1. Nanoparticles of Diamond (NDs)

**Figure 5:**
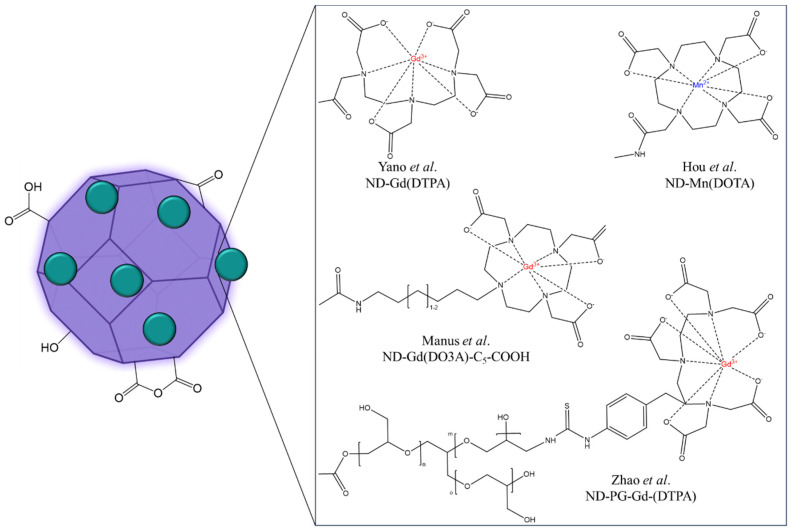
Structures of some ND-based contrast agents discussed in this work (ND-Gd(DO3A) [26,27] ND-Gd(DTPA) [28] ND-Mn(DOTA) [29]; and ND-PG-Gd(DTPA) [30]).

Nanodiamonds, known for their biocompatibility and dispersibility in water, can be used for both fluorescence and MR imaging [19,20,21,22]. A typical magnetic resonance diagnostic approach using nanostructured diamond particles relies on functionalizing the particle surface with covalently grafted lanthanide. Nanosized diamond particles (so-called nanodiamonds) are synthesized in large-scale production via a detonation procedure where a trinitrotoluene and hexogen (TNT/RDX) mixture is detonated under an oxygen-deficient atmosphere [21,23,24]. The isolated detonation nanodiamond (DNDs) particles, reportedly 4–5 nm in diameter size, exhibit both a good metabolic stability, since they are composed of mostly *sp*^3^-hybridized carbon atoms, and an inherent surface chemistry, with a variety of functional groups which can be oxidized into carboxylic acid functions [20,25]. This rich surface chemistry directly allows for the covalent grafting of paramagnetic chelates (Figure 5), creating a diamond-based platform detectable by conventional high-field MR imaging.

**Figure 6:**
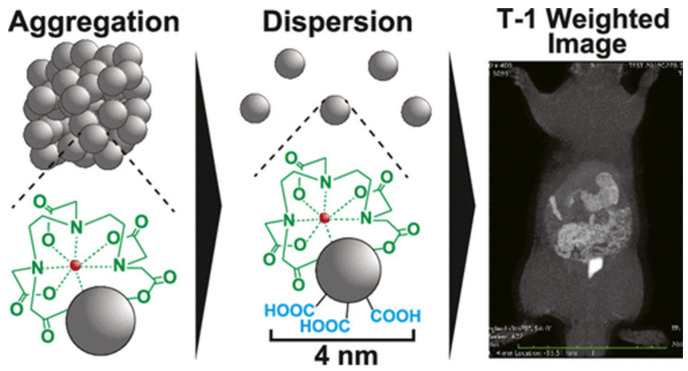
Illustration of ND-Gd-DTPA developed by Yano et al. Reprinted with permission from [28]. Copyright 2021 American Chemical Society.

One of the first examples of a gadonanodiamond (Gd-ND) platform, developed by Manus and coworkers [26], involved the conjugation of Gd^3+^-DO3A-amine onto carboxylated DNDs using peptidic coupling. This innovative approach resulted in a remarkable ten-fold increase in longitudinal relaxivity (r_1_ of 58.8 ± 1.2 mM^−1^ s^−1^ per Gd^3+^ at 1.41 T, 37 °C). This improvement can be attributed to greater molecular tumbling rates (optimized τ_M_ value), surpassing that of the “free-motion” current Gd-DO3A contrast agent (r_1_ value of 5.4 ± 0.2 mM^−1^ s^−1^). This group reported the first T_1_-weighted MRI phantom images obtained within a micromolar range [26]. The hydrodynamic diameter (D_H_) was increased from 21 to 128 nm after the surface conjugation step, suggesting the aggregation of gadolinium-grafted DNDs, which contributed to the remarkable increase in longitudinal relaxivity. Viability studies on human ovary adenocarcinoma (SKOV-3) cells revealed no significant cytotoxic effect. This group presented in vivo longitudinal MRI monitoring experiments (7 T) for tracking cancer growth, morphology, and differentiation [31]. Subsequently, a second Gd-ND generation was designed through the grafting of Gd-DO3A-C_5_-COOH chelate (r_1_ = 6.4 s^−1^ mM^−1^, 1.41 T, 37 °C) onto amino-functionalized DNDs as aggregates [31]. A two-fold Gd^3+^ content increase (~ 1.5 μmol_Gd_/mg of DND aggregates) was obtained compared to the first generation. Strikingly, as observed in their water proton nuclear magnetic relaxation dispersion profile, the r_1_ of Gd-ND (D_H_ = 75 nm; r_1_ = 11.5 s^−1^ mM^−1^, 7 T) surpassed the current chelates across all field strengths, however, with a similar pattern even in the 0.5–2 T range, which may have been due to the retention of rotational freedom even after grafting onto ND (similar τ_R_ values). In vivo MRI (26 days of monitoring) of a mouse bearing a gadonanodiamond-labelled xenograft showed a 165% enhancement in r_1_ and confirmed the viability of the Gd-ND system for MRI and cell tracking [27]. In addition, Nakamura et al. [32] and Yano and collaborators [28] reported the fabrication of ND-Gd(DTPA) systems by condensing a Gd-DTPA contrast agent onto nanodiamonds with hydroxyl groups or preoxidized carboxylic terminations, respectively. Regarding their first surface strategy, microscale aggregates were observed in water, whereas carboxylated NDs proved to be an efficient platform for condensing gadolinium chelates (Figure 6).

The Gd^3+^ content reached around 94 mg/g, as estimated by ICP-AES, however, the relaxivity was not specified. They evaluated the platform (D_H_ = 630 nm, PBS) using low-field MRI (1.5 T) for a lymphatic system evaluation. Subsequently, their as-synthesized DND-Gd(DTPA) aggregates exhibited good dispersion in high-ionic-strength media (PBS) and showed a high lymphatic T_1_-weighted MR image intensity. This demonstrated the considerable potential of the system as an MRI contrast agent for tracking cancer cell growth in in vivo evaluations. Alternatively, another way to ensure a paramagnetic coating onto nanodiamonds consists of the direct grafting of gadolinium ions via ion exchange between hydrogen atoms from oxygen-containing groups such as carboxyl functions and Gd^3+^ from gadolinium nitrate in aqueous media (Gd(NO_2_)_3_·6H_2_O) [33,34]. According to the study developed by Panich et al. [34], Gd^3+^ ions were chemically bonded to the particle surface and were, thus, able to interact with both protons and electron spins from the nanodiamonds particles. The distance between the metal ion and the particle surface was approximately 0.32 nm, as estimated by ^13^C NMR. This proximity ensured a high paramagnetic spatial density (18 Gd^3+^ ions per 5-nm DND particle; roughly 3.28%_wt_.) [33,34]. This density enables for effectively influencing the spin relaxation of paramagnetic centers within the DND, such as NV^-^ color centers and dangling bonds on nearby water molecules. The developed ND-Gd^3+^ systems exhibited a hydrodynamic diameter D_H_ of approximatively 7 nm (DLS measurements in volume intensity), with an r_1_ of 33.4 s^−1^ mM^−1^ (8 T, 37 °C) [34,35]. However, subsequent surface modifications (i.e., polymer-stabilized coating) were necessary to prevent bundling of the gadonanodiamonds through Van Der Waals interactions. While very high proton relaxivities induced by these nanostructures are evidenced, careful solubility and stability considerations must be evaluated. In this framework, Panich et al. [36] further developed polyvinyl pyrrolidone (PVP)-coated DNDs grafted with Gd^3+^ as a potentially safer probe for in vivo MRI applications. As a compromise over nonstable systems in saline media, a lower r_1_ = 15.9 s^−1^ mM^−1^ was reached for a DND-Gd^3+^/PVP coated gadolinium-grafted DND system (2–4 Gd^3+^ ions per particle), although the coating agent hindered the access of water molecules nearby paramagnetic ions [36] and showed a dose-dependent T_1_-weighted signal. Furthermore, in that context, Zhao and collaborators [30] designed a nanodiamond-hyperbranched polyglycerol-gadolinium(III) conjugate through multistep organic transformations to enhance its dispersibility and stability in biological media with good parameters over three months (D_H_ = 51/50 nm in water/PBS, respectively). The DND-PG-Gd-DTPA system carried 22.4 µg_Gd_/mg of DND and was covered by a polyglycerol coating with a thickness of approximatively 10–15 nm. The relaxometric measurement of the stabilized system (18 nm) was significantly higher than that of clinical Gd-DTPA (r_1_ = 19.4 mM^−1^ s^−1^ vs. 3.7 mM^−1^ s^−1^ at 1.5 T). Other paramagnetic complexes have also been extensively studied as T_1_ agents for MRI; within this context, Hou and co-workers [29] constructed different functionalized nanodiamonds with manganese (Mn^2+^) chelating agents attached onto DNDs (DND-Mn(EDTA) or ND-Mn(DOTA)). The resulting Mn-labeled platforms, characterized by a Mn^2+^ loading efficiency of 4 nmol/mg_DND_, showed an approx. 13-fold enhancement in r_1_ value (r_1_ = 22.7 mM^−1^ s^−1^; Mn-EDTA, r_1_ = 1.7 mM^−1^ s^−1^ at 7 T). The probe was used as dual agent for enhanced liver tumor diagnosis by manganese conjugation onto DNDs. Similarly to the systems developed by Panich et al. [34], Mn-based nanodiamonds (0.52 grafted Mn^2+^ ion per particle) were described, for which the DND-inherent paramagnetic contribution was subtracted from the water proton spin-lattice R_1_, leading to an r_1_^Mn^ = 19.6 s^−1^ mM^−1^ (r_1_^DND^ = 2.1 s^−1^ mM^−1^ (8 T, 37 °C) per Mn^2+^ ions [37]. Clear evidence for the chemical bonding of Mn^2+^ ions to the DND surface was established. To ensure colloidal stability in physiological media, the system was coated using PVP, which resulted in a slight decrease in the relaxivity (r_1_ = 17.6 s^−1^ mM^−1^) as a compromise over better colloidal behavior [37,38]. Shan et al. [39] described the covalent grafting of 2-thenoyltrifluoroacetone (TTA) triethoxysilane derivatives complexes coordinated with rare-earth (RE = Gd^3+^ and Eu^3+^) ions for paramagnetic and fluorescence properties, respectively. Their resulting ND-RE(TTA) system (mean core size of 4.2 nm) as agglomerates exhibited T_1_-weightened MRI features (r_1_ = 1.08 s^−1^ (mg/mL)^−1^ at 3 T, normalized per molar concentration of ND-RE(TTA)) and a strong red emission (613 nm), considering the coordinated ions [39]. In addition, their rare-earth functionalized ND platform can be combined with imaging and has a drug delivery capability; the ND-RE(TTA)/DOX system demonstrated an efficient drug storage capability with doxorubicin (DOX, anticancer drug; 375 μg_DOX_.mg^−1^) and showed significant pH-dependent drug release behavior. In vitro/in vivo experiments demonstrated its high potential for chemotherapy towards gastric cancer cells in comparison to ND-RE(TTA) [39]. Alternatively, Niu et al. [40] designed an “in core” paramagnetic Mn-based material; Mn^2+^ ions were incorporated inside the diamond core, generated by high-dose ion implantation on larger NDs obtained from the mechanical grinding of high-quality HPHT (high-pressure and high-temperature) diamond microcrystals (150 nm in mean nominal core). The Mn^2+^-doped@ND system (Mn ions content: 1.65%_wt_.) is able to interact with surrounding water molecules to enhance relaxivity (r_1_ = 0.11 s^−1^ (mg/mL)^−1^; r_1_^ND^ = 0.02 s^−1^ (mg/mL)^−1^ at 7 T) as a contrast enhancement probe for the in vitro/in vivo MR imaging of cancer tumors in mice. Since the interaction between paramagnetic ions and protons from surrounding water molecules is not a direct process, the relaxivity is highly variable according to the surface proximity of Mn^2+^ ions. Similarly, an iron-doped HPHT-ND was obtained by iron implantation and showed the capabilities of nanodiamonds as attractive *T*_2_ contrast agents (r_2_^Fe@ND^ = 0.951 mL s^−1^ g^−1^ vs. r_2_^ND^ = 0.145 mL s^−1^ g^−1^, 7 T) [41]. Furthermore, we can mention that a particularity towards nanodiamonds was reported by Waddington and collaborators in their applications as MRI contrast agents without any lanthanide complexations using ultra-low magnetic fields [42,43,44]. Briefly, intrinsic paramagnetic structural defects and impurities (overall content: 6 × 10^19^ spin/g) on nanodiamond surfaces can be used to transfer spin polarization to the proton spin nuclei of bulk water molecules. This strategy generates high-contrast MRI images relying on the in situ hyperpolarization technique through Overhauser-enhanced MRI (OMRI) with a contrast sensitive to NDs’ concentration. However, this technology may suffer from a low signal-to-noise ratio associated with a low/ultra-low magnetic field (6.5 mT). For high-field MRI evaluation, attempts have been made towards an alternative to gadolinium-free contrast agents or hyperpolarization techniques using paramagnetic centers [45]. Lazovic et al. [45] investigated the relaxometric characteristics of DND after air-oxidized treatment and observed an increase in the relaxivity rate when comparing the values before and after the air oxidation process (r_1_ = 1.8 vs. 11.3 s^−1^ mM^−1^ at 7 T after oxidation) [45]. 

**Table 1:** Summary of modified nanostructures for biolabeling applications as potential T_1_-weighted contrast agents for MRI, with relaxivity values normalized per paramagnetic ion concentration. “u.d.”: stands for unspecified data; TEM: transmission electron microscopy; AFM: atomic force microscopy; “Ln@material”: stands for “lanthanide/transition metal-containing material as species (ion or molecule) coated by a specific material; DND: detonation nanodiamonds; HPHT-ND: high-pressure high-temperature milled ND; DLS: dynamic light scattering for hydrodynamic diameter (D_H_) evaluation; PVP: polyvinylpyrrolidone as coating agent; PCTA: pyridine containing triAza; 3,6,9,15-tetraazabicyclo[9.3.1]pentadeca-1(15),11,13-triene-3,6,9-triacetic acid. a: volume intensity; b: number intensity; c: lateral dimension.

Material	Systems	Size (TEM) (nm)	r_1_^para^ (s^−1^ per mM Paramagnetic Center Mn/Gd) in Water or Buffer	Enhancement (%)	Magnetic Field (Tesla)	Ref.
*Nanodiamond*	DND-C_6_-Gd(DO3A) *q* = 2	128 nm (DLS)	58.8 s^−1^ mM^−1^ Gd-DO3A: 5.4 s^−1^ mM^−1^	988%	1.4 T/37 °C	[26]
	DND-C_5_-Gd(DO3A) *q* = 2	75 nm (DLS)	11.1 s^−1^ mM^−1^ Gd-DO3A-C_5_-COOH: 6.4 s^−1^ mM^−1^ 11.5 s^−1^ mM^−1^ Gd-DO3A-C_5_-COOH: 4.8 s^−1^ mM^−1^	73% 139%	1.4 T/37 °C 7 T/37 °C	[31]
	DND-Gd(III)	7 nm (DLS)^a^	33.4 s^−1^ mM^−1^ Gd-BOPTA: 4.8 s^−1^ mM^−1^	596%	8 T/37 °C	[35]
	DND-PG-Gd(DTPA) (PG: polyglycerol) *q* = 1	18 nm	19.4 s^−1^ mM^−1^ Gd-DTPA: 3.7 s^−1^ mM^−1^ 16.7 s^−1^ mM^−1^ Gd-DTPA: 3.5 s^−1^ mM^−1^ 8.2 s^−1^ mM^−1^ Gd-DTPA: 3.4 s^−1^ mM^−1^	424% 377% 141%	1.5 T 3 T 7 T	[30]
	ND-Gd(III)/PVP	45–70 nm (DLS)^a^	15.9 s^−1^ mM^−1^ ND-Gd(III): 33.4 s^−1^ mM^−1^	−52%	8 T/37 °C	[36]
	DND-Gd(DTPA) *q* = 1	4.2 nm 630 nm (DLS)	u.d.	_	1.5 T	[28]
	DND-Mn(EDTA)	65 nm (DLS)	22.7 s^−1^ mM^−1^ Mn-EDTA: 1.7 s^−1^ mM^−1^	1235%	7 T	[29]
	DND-Mn(II)	4.5 nm	19.6 s^−1^ mM^−1^ DND: 2.1 s^−1^ mM^−1^	833%	8 T/37 °C	[37,38]
	DND-Mn(II)-PVP	4.5 nm	17.6 s^−1^ mM^−1^ DND: 2.1 s^−1^ mM^−1^	738%	7 T	[29]
	DND (air oxidized)	3–4 nm	11.3 s^−1^ mM^−1^ DND: 1.7 s^−1^ mM^−1^	558%	7 T	[45]
	Mn(II)-doped@HPHT-ND	150 nm 1 µm (DLS)	0.11 s^−1^ (g/mL)^−1^ ND: 0.02 s^−1^ (g/mL)^−1^	450%	7 T	[40]
	Fe-doped@HPHT-ND *(T_2_ agent)*	100 nm	r_2_^Fe@ND^ = 0.95 s^−1^ (mg/mL)^−1^ r_2_^ND^ = 0.14 s^−1^ (mg/mL)^−1^	555%	7 T	[41]
	ND-RE(TTA)/DOX *(RE: rare-earths = Eu^3+^, Gd^3+^)* *(TTA: 2-thenoyltrifluoroacetone complexes)*	4.2 nm	r_1_ = 1.1 s^−1^(mg/mL)^−1^	_	3 T	[39]
*Carbon nanotube*	MWCT-Gd(DTPA)	20–30 nm (in diameter size) 0.5–2 µm (in length size)	6.61 s^−1^ mM^−1^ Gd-DTPA: 2.1 s^−1^ mM^−1^	314%	7 T	[46]
	MWCT-Gd_2_O_3_	10–20 nm (in diameter size) (SEM)	18.9 s^−1^ mM^−1^ Gd_2_O_3_ NPs: 9.9 s^−1^ mM^−1^	91%	9.4 T/25 °C	[47]
	Gd(III)-doped-US-SWCT	20–80 nm (in length size)	170 s^−1^ mM^−1^ Gd-DTPA: 4 s^−1^ mM^−1^	4150%	1.41 T/40 °C	[48]
	Gd(III)-doped-US-SWCT/PLGA	20–80 nm (in length size)	r_2_ = 578 s^−1^ mM^−1^	_	7 T/25 °C	[49]
*C_2n_-like fullerene*	Gd^3+^@C_60_[C(COOH)_2_]_10_ *(endofullerene)*	10 nm (DLS)	4.6 s^−1^ mM^−1^	_	0.5 T/40 °C	[50]
	Gd^3+^@C_60_[C(COOH)_2_]_10_ *(endofullerene)*	u.d.	10.4 s^−1^ mM^−1^ Gd^3+^@C_60_(OH)_x_: 38.5 s^−1^ mM^−1^	−73%	0.5 T/25 °C	[51]
	Gd^3+^@C_60_(OH)_x_ *(endofullerene)*	u.d.	38.5 s^−1^ mM^−1^ Gd^3+^@C_60_[C(COOH)_2_]_10_: 10.4 s^−1^ mM^−1^	270%	0.5 T/25 °C	[51]
	Gd^3+^@C_82_[OH]-FA/FITC *(endofullerene)*	127 nm (DLS)	20.2 s^−1^ mM^−1^ Gd-DTPA: 4.5 s^−1^ mM^−1^	348%	0.5 T/37 °C	[52]
	C_60_-PEG-Gd(DTPA)	u.d.	5.1 s^−1^ mM^−1^ Gd-DTPA: 5.3 s^−1^ mM^−1^	−4%	7 T/25 °C	[53]
	C_60_-Gd(DOTA)	8.9 nm (DLS)^b^	49.7 s^−1^ mM^−1^ Gd-DOTA: 5.4 s^−1^ mM^−1^ 29.2 s^−1^ mM^−1^ Gd-DOTA: 3.2 s^−1^ mM^−1^	820% 812%	0.5 T 1.5 T	[54]
	Gd_3_N@C_80_[DiPEG_350Da_(OH)_x_] *(endofullerene)*	75 nm (DLS)	75.7 s^−1^ mM^−1^ 79.0 s^−1^ mM^−1^ 22.7 s^−1^ mM^−1^	_	0.35 T/25 °C 2.4 T/25 °C 9.4 T/25 °C	[55]
	Gd_3_N@C_80_[DiPEG_5kDa_(OH)_x_] *(endofullerene)*	37 nm (DLS)	46.3 s^−1^ mM^−1^	_	2.4 T/25 °C	[54]
	Gd_3_N@C_80_-ZD2peptide *(endofullerene)*	2.8 nm	74.6 s^−1^ mM^−1^ Gd_3_N@C_80_: 57.1 s^−1^ mM^−1^ 24.8 s^−1^ mM^−1^ Gd_3_N@C_80_: 24.7 s^−1^ mM^−1^	30% 0.4%	1.5 T 7 T	[56]
	Gd_3_N@C_80_-DOX-RNPs *(endofullerene)*	146 nm (DLS) pH = 7.4	17.8 s^−1^ mM^−1^ 5.7 s^−1^ mM^−1^	212%	7 T/pH = 6.6 7 T/pH = 7.4	[57]
	C_60_-Gd_2_(DOTA)_2_	171 nm (DLS)	18.2 s^−1^ mM^−1^ Magnevist: 4.7 s^−1^ mM^−1^	287%	4.7 T/20 °C	[58]
	C_60_-Mn(APTSPP)	76 nm	19.2 s^−1^ mM^−1^ Mn(APTSPP): 11.3 s^−1^ mM^−1^ 12.2 s^−1^ mM^−1^ Mn(APTSPP): 8.2 s^−1^ mM^−1^	70% 49%	0.5 T/37 °C 3 T/37 °C	[59]
*Graphene oxide*	GO-Gd(III)	36–44 nm (AFM)	78–85 s^−1^ mM^−1^	_	1.41 T/37 °C	[60]
	GO-Gd(DTPA) *q* = 1	76 nm	28–32 s^−1^ mM^−1^	_	0.5 T/37 °C 3 T/37 °C	
	GO-Gd(DO3A) *q* = 2	36–44 nm (AFM) < 50 nm (SEM) 20–50 nm (AFM)^c^	49–63 s^−1^ mM^−1^	_	1.41 T/37 °C 1.5 T 11.7 T	
	rGO-Gd(DTPA) *q* = 1		16.8 s^−1^ mM^−1^ Gd-DTPA: 4 s^−1^ mM^−1^	321%		
	GO-PEG-Gd(DOTA)		14.2 s^−1^ mM^−1^ Gd-DOTA: 4.5 s^−1^ mM^−1^	215%		
	G-Mn(DPDP)	±100 nm	92 s^−1^ mM^−1^ Mn-DPDP: 2.8 s^−1^ mM^−1^	3185%	0.5 T	[61]

Therefore, summarizing the use of nanodiamonds as a support in T_1_-weightened contrast enhancement for MRI, this diagnostic approach can be achieved by commonly functionalizing the oxygenated or aminated nanodiamond surface with modified lanthanide (Gd or Mn) chelates. In addition, polymer chains can be linked onto the surface to ensure biostability and monodispersity. Beyond lanthanide-based coating agents, there is a trend of the use of diamond-like nanoparticles as carriers themselves due to paramagnetic defects that are able to generate a contrast [45,62,63].

### 3.2. Carbon Nanotubes (CNTs)

**Figure 7:**
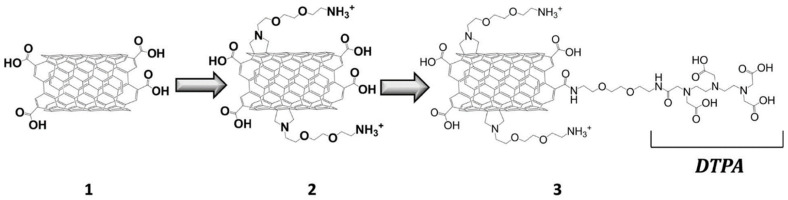
Illustration of DTPA-functionalized MWNT developed by Servant et al. (Reprinted with permission from [46]). The carboxylated MWNT (1) are modified through 1,3 dipolar cycloaddition in order to introduce amine functions (2), which then react with DTPA via peptidic chemistry (3).

CNTs are classified into single-walled carbon nanotubes (SWCTs) and multi-walled carbon nanotubes (MWCTs), according to the number (single or multiple) of graphene sheets that form the cylindrical tube. This material is produced using electric arc discharge, laser ablation, or chemical vapor deposition. From a structural point of view, CNTs are composed of fullerene hemisphere (tips) and curved graphite (sidewalls). Besides CNTs’ applications as drug delivery systems [64], various researchers have focused on biomedical imaging applications of functionalized carbon nanotubes [65,66,67]. Similarly to gadonanodiamond, carbon nanotubes have been chelated directly with Gd^3+^ chelates (referred to as gadonanotubes). Servant and co-workers [46] developed a gadolinium-functionalized MWNT as a T_1_-weighted contrast agent for MRI cell labelling and tracking. Their system was covalently functionalized using a conventional gadolinium chelating agent (DTPA) grafted onto the acid-treated nanotube surface (Figure 7).

The oxidation step enables the generation of hydroxyl and carboxyl groups either on sidewall defects or at the open ends to enhance dispersibility in aqueous media. Therefore, the chemical functionalization of CNTs is assumed through covalent attachment on these sidewalls. Their T_1_ relaxivity (r_1_ = 6.6 s^−1^ mM^−1^) was three-fold greater than that of Magnevist^®^ (r_1_ = 2.1 s^−1^ mM^−1^) at a magnetic field of 7 Tesla, showing a promising T_1_ contrast enhancement in vitro. Notably, gadonanotubes have NMRD relaxation profiles characteristic of gadolinium ions coordinated to a slowly tumbling environment [68]. Ultra-short (US) CNTs (known as US-tubes) have already been recognized as high-performance T_1_-weighted MRI contrast probes when internally loaded with paramagnetic centers (i.e., Gd^3+^ ions). Sitharaman reported a three-fold greater longitudinal relaxivity of gadonanotubes (r_1_ = 170 mM^−1^ s^−1^) [48,49]. Subsequent surface modification using a PLGA polymer nanocomposite leads to a system characterized by an r_2_ of 578 mM^−1^ s^−1^ at 7 T (25 °C) [49]. Alternatively, SWNT (<100 nm) with a negligible metal content (< 1%_wt_. Fe) showed a superior T_2_ relaxation efficiency (r_2_/r_1_ = 5.6) as one of the most promising candidates for advanced T_2_-weightened applications such as molecular and cellular imaging using MRI [69]. Alternatively, a paramagnetic CNT doped with gadolinium oxide NPs was designed by the NPs’ deposition onto the carbon allotrope in order to decrease the Gd^3+^-based toxicity. In addition, their MWCNT/Gd_2_O_3_ hybrid nanostructure showed a significant increase in longitudinal relaxivity (r_1_ = 18.93 s^−1^ mM^−1^ at 9.4 T, 25 °C) [47].

### 3.3. Buckminsterfullerene (C_2n_)

**Figure 8:**
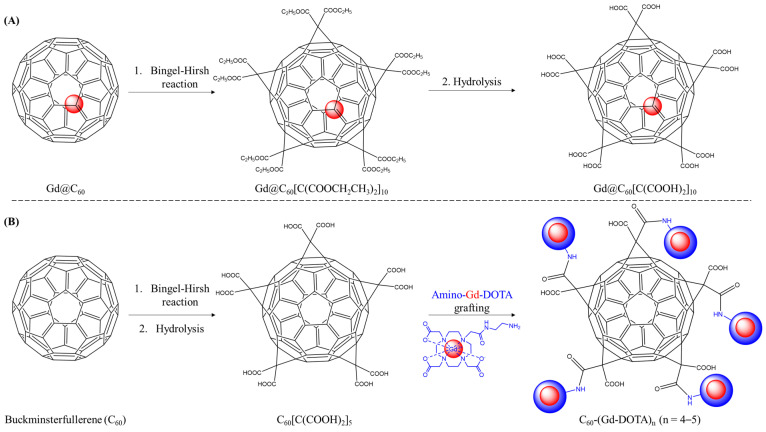
Illustration of the two general strategies for the preparation of water-soluble gadofullerenes derivatives as MRI contrast agents. (**A**) Strategy for the preparation of endohedral gadofullerenes, as described by Bolskar et al. [50]; (**B**) strategy for the preparation of surface-modified fullerenes, as described by Wang et al. [54].

Two different fullerene architectures are known: empty ones and endohedral fullerenes (or endofullerenes), which are carbon cages filled with atoms or molecules. These cage-like structures exhibit a high potential and versatile biological applications in biomedicine, such as antioxidants, antibacterial activity, enzyme inhibition agents, biosensors, or as MRI contrast agents [70,71]. For the latter, water-soluble Gd@C_2n_ derivatives are common endo-metallofullerenes consisting of a large cage-like structure based on a closed sheath of *2n* carbon atoms entrapping a gadolinium atom. Covalent derivatization of the outer non-hydrophilic surface is used to overcome the native insolubility of the buckminsterfullerene carbon allotrope; hydroxyl and carboxyl terminations are the two main functional groups that have been used to provide aqueous-soluble C_2n_ materials for biological applications. Two strategies in the design of paramagnetic structures based on water-soluble fullerenes are summarized in Figure 8.

One of the first water-soluble Gd@C_60_ generated as an MRI contrast agent was assembled by Bolskar and collaborators [50] in 2003, starting from carbon arc synthesis after entrapping a gadolinium ion (Gd^3+^, atomic radius: 180 pm) during sublimation transition. HPLC purifications are generally used to extract fractions of arc discharge products. Next, the Bingel–Hirsh reaction enabled the formation of a Gd@C_60_[C(COOCH_2_CH_3_)_2_]_10_ intermediary following its hydrolysis with sodium hydride to produce a polycarboxylated water-soluble fullerene derivative, the Gd@C_60_[C(COOH)_2_]_10_. Its relaxivity was comparable to the values of clinically approved gadolinium chelates at a proper clinical magnetic field; moreover, by capturing a Gd^3+^ ion in the C_60_ cage, there is no risk of release or transmetallation [72]. In contrast with gadonanodiamond clusters from Rammohan et al. [27], the NMRD profiles of gadofullerene show a bump around 0.47–1.41 T as a result of slow rotational motion and a long rotational correlation time (*τ_R_* = 2.6 ns) [51]. In such a system, the bulk water molecule cannot interact directly with the paramagnetic center, since direct Gd^3+^⋯H_2_O bonding is not accessible (no inner-sphere coordinated water). Sitharaman et al. [73] suggested a greater impact on the outer-sphere relaxivity attributed to the motion constraint of aggregates, as observed in polycarboxylated (Gd@C_60_(COOH)_x_) vs. polyhydroxylated endofullerene (Gd@C_60_(OH)_x_) systems [51,73,74]. As a result, the relaxation contribution occurs through a chemical exchange between protonated species at the fullerene surface (COOH or OH groups) and the bulk water molecules. Furthermore, surface modification inducsd differences in the type and number of groups on the fullerenes outer surface, which results in different relaxivities [74]. An overview of a similar study can be found in a chapter from Kumar et al. [72]. Briefly, the group discussed high relaxivity values, particularly exemplified by Gd_3_N@C_80_(di-PEG5k)(OH)_x_ material, which exhibited an r_1_ value of 85 s^−1^ mM^−1^. The high relaxivity of aggregated gadofullerenes is attributed to the rapid exchange of water molecules with the bulk, while a decrease in relaxivity was observed upon disaggregation, typically from 700 to 79 nm, as seen in Gd@C_60_[C(COOH)_2_]_10_, for instance [72]. Various investigations have been conducted to analyze the variations in the preparations of promising systems, including the degree of hydroxylation/carboxylation, particles sizes, agglomeration state, buffered conditions, pH changes, or magnetic field and temperature [72]. Additionally, surface modification with vectors has also been studied; a work by Zheng et al. [52] reported the construction of a dual molecular imaging tracer based on an endofullerene nanoplatform for the early diagnosis of folic acid receptor (FAR) over-expressed in tumors. For this purpose, they used Gd@C_82_ paramagnetic endofullerenes containing hydroxyl and carboxylic acid groups on the external surface to integrate multimodality via the fluorescent 5-FITC cadaverine (5-FITC, a fluorescein isothiocyanate, abs./em.: 492/516 nm, 5-((5-aminopentyl)thioureidyl) fluorescein) and targeting with the folic acid recognition moiety. Its receptor (FAR) is over-expressed in cancerous human cells (i.e., pathological cells in lungs, kidney, and breast), therefore, conjugation of the vitamin to its receptor ensured the specificity and the selectivity of diagnoses. Their model suggested a high potential as dual optical/MR imaging probes, since the endohedral metallofullerene nanoplatform exhibited an increased r_1_ value reaching 20.2 s^−1^ mM^−1^ at 0.5 T (Gd-DTPA; r_1_ = 4.5 s^−1^ mM^−1^). Compared with previously explored paramagnetic endofullerenes as MR contrast agents, we can note that the longitudinal relaxivity between Gd@C_60_[C(COOH)_2_]_10_ (r_1_ = 4.6 s^−1^ mM^−1^, 0.5 T, 40 °C, D_H_ = 10 nm) [50] and Gd@C_82_(OH)_40_FA/FITC (r_1_ = 20.2 s^−1^ mM^−1^, 0.5 T, 37 °C, D_H_ = 127 nm) [52] noticeably depends on the cage outer surface nature. Indeed, most of the water-soluble fullerene tends to aggregate in aqueous solution to form clusters with sizes related to the functional groups on the carbon cage, which does have an impact on the relaxation efficiency by affecting $\tau_{R}$. For both systems, DLS measurements were performed to determine the aggregation state, and, in the case of Gd@C_82_(OH)_40_FA/FITC, an increase in the relaxivity was observed as a result of the formation of aggregates (characterized by a hydrodynamic diameter of 127 nm). Liu’s team explored a paramagnetic PEGylated fullerene derivative (C_60_-PEG-DTPA-Gd(III)) as an MRI contrast agent, in which gadolinium complexes were introduced on the PEG terminal group [53]. Coupled with gadolinium-DTPA, their structure was used as a theranostic tool, since these C_60_ cages are able to generate superoxide anions upon light irradiation to eradicate cancerous cells [53]. Intravenous administration of the agent to mice affected by cancer was followed by MRI, and a tumor mass was observed due to accumulation into the affected tissue efficacy. The timeline of the C_60_-PEG-DTPA-Gd(III) signal increased in comparison to that induced by the Magnevist^®^ and light irradiation showed a significant tumor photodynamic therapy effect under appropriate conditions. For gadofullerenes and gadonanotubes, a second sphere mechanism played a key role in their relaxation properties. More recently, another group focused on an empty C_60_ cage with paramagnetic surface functionalization to increase the relaxivity value; Wang et al. [54] prepared a malonic acid modified-C_60_ nanostructure according to the protocol used by Bolskar. Amino-Gd-DOTA complexes were then attached onto the C_60_ external surface to design the C_60_(Gd-DOTA)_n_ (4 to 5 Gd^3+^ chelates per fullerene, according to mass spectrometry analysis). The relaxivity (r_1_ = 79 s^−1^ mM^−1^, 2.4 T, 25 °C) increased by a factor of nine compared to Gd-DOTA, however, these structures could not significantly increase the number of coordinated water molecules. In cause, the carboxyl groups (10 units) did not participate in the relaxation processes due to steric hindrance. Note that a longitudinal relaxivity r_1_ of 18.2 s^−1^ mM^−1^ was obtained for a C_60_(Gd-DOTA)_2_ system at a magnetic field of 4.7 T (20 °C) [58]. Alternatively, Zou and coworkers reported a manganese-porphyrin (APTSPP) compound grafted to a C_60_ fullerene (r_1_ = 19.2 s^−1^ mM^−1^, 0.5 T, 37 °C) [59], with half the relaxivity of gadofullerene (C_60_-Gd(DOTA); r_1_ = 49.7 s^−1^ mM^−1^, 0.5 T) [54]. Note that, during the synthesis of the C_2n_-based structure, one to three Gd^3+^ ions can be trapped inside the fullerene cage. In this context, to increase the r_1_ for T_1_-weightened MRI, an endohedral trimetallic nitride metallofullerene derivative is introduced during the synthesis step. In addition, for in vivo applications, gadofullerene can be commonly physisorbed using PEGylated-hydroxylated terminations. Zhang et al. exploited Gd_3_N@C_80_[DiPEG_n_(OH)_x_] systems with four different molecular weight PEG chains [55]. These PEG-derivative materials showed a much higher r_1_ relaxivity in comparison to conventional Gd-based contrast agents, however, r_1_ tended to decrease as the hydrodynamic size became greater, as a result of the aggregation state. Typically, r_1_ values of 79.0 s^−1^ mM^−1^ and 46.3 s^−1^ mM^−1^ were obtained for the PEG_350_-coated (D_H_ = 95 nm) and PEG_5000_-coated (D_H_ = 37 nm) systems, respectively, at 2.4 T (25 °C) [55]. Interestingly, Han et al. synthesized a high-relaxivity targeted platform by conjugating a small linear ZD2 peptide onto trimetallic endo-gadofullerenes C_80_ (three Gd^3+^ per particle) [56]. The nanosystem was designed for the sensitive imaging of extradomain-B fibronectin in aggressive breast cancer tumors. The observed r_1_ relaxivity had value about 10 times higher than Gd-based conventional chelates (i.e., Gd-DTPA, Gd-HP-DO3A), which reached 74 s^−1^ mM^−1^ per Gd^3+^ ion at 1.5 T for the nanosystem. Moreover, its relaxivity was higher than that observed on the starting nonvectorized material system (Gd_3_N@C_60_) due to the slower tumbling rate and τ_R_ decrease correlated with the larger size. Interestingly, Wang et al. [57] designed an activatable MRI contrast agent for tumor signal amplification while affording monitoring for drug doxorubicin (DOX) release (Gd_3_N@C_80_-DOX-RNPs). Herein, an endofullerene was encapsulated into pH-responsive polymer nanoparticles (RNPs) based on PEG chains. At physiological pH (pH = 7.4), both Gd_3_N@C_80_ and DOX were entrapped within the polymer-modified nanostructures, shielding from the aqueous environment; therefore, the system can be characterized by a relatively low relaxivity (r_1_ = 5.7 s^−1^ mM^−1^; pH = 7.4) and low drug release. Strikingly, in acidic tumor microenvironments (pH = 6.6), the conversion of the pH-responsive polymer leads simultaneously to an increase in the S/N ratio in MRI (r_1_ = 17.8 s^−1^ mM^−1^; pH = 6.6) and a faster drug release [57]. Hence, an accurate tumor diagnosis is obtained, and the treatment monitoring can be studied with a low risk of Gd^3+^ release. 

### 3.4. Graphene and Graphene Oxide Nanosheet (GO)

Oxidized derivatives of graphene, graphene oxides, are two-dimensional nanosheets containing various oxygenated functional groups (hydroxyl, carboxyl, and epoxy groups). Graphene and its derivatives serve as nanocarriers or platforms onto which (para)magnetic agents are anchored. This is attributed to their high specific surface area, which prevents the aggregation of these CNMs, providing additional stability and a significant MRI efficiency [75]. Zhang and co-workers exploited an outer strategy to design a two dimensional ultra-small graphene oxide construct (30 nm in size) agent for T_1_-MRI applications [76]. They first synthesized ultra-small GO nanosheets from graphite using a modified version of Hummer’s method [77] and simultaneously stabilized and functionalized their material with branched PEG (six armed-amine PEG, 10 kDa), then conjugated a Gd-DOTA complex with amine-terminated PEG. Their resulting Gd(III)-labelled graphene oxide was three times as efficient (r_1_ = 14.2 s^−1^ mM^−1^) compared to the commercialized low-molecular-weight one (Gd-DOTA; r_1_ = 4.5 s^−1^ mM^−1^, 11.7 T) [76]. In addition, their system was suitable for the in vitro and in vivo labeling of human mesenchymal stem cells, leading to an improved cellular MRI effect without noticeable adverse effects on the proliferation of the cells. Their work demonstrated the promising application of their agent for stem cell labeling, which may find a purpose in stem cell therapies for regenerative medicine. Meade et al. [60] studied a gadographene library encompassing two different Gd chelates (DTPA and DO3A). Strikingly, Gd(III) associated with these CNTs represents a potential break-through in sensitivity for NMR applications and deals with the theoretical limit for a single bound (*q* = 1) water molecule. Longitudinal r_1_ and transverse r_2_ values are within the 12–85 s^−1^ mM^−1^ and 24–115 s^−1^ mM^−1^ ranges, respectively, depending on the Gd chelating agent and the carbon backbone. Based on an NMRD analysis, this large relaxivity is attributed to the modified Florence model theory incorporating the Lipari–Szabo relaxation approach (τ_R_ > 1000 ns and τ_M_: 80–110 ns) [60]. Kanakia et al. [61] studied the in vitro and in vivo relaxivity of their system G-Mn(DPDP) (92 s^−1^ mM^−1^ at 0.5 T; chelate brand name: Teslascan^TM^) while studying the dose efficiency in a rodent model for high-magnetic-field MR imaging. The magnetic resonance contrast enhancement was correlated with the close proximity of protons in the gadographene system and with the fast exchange in between. The formulation at all dosages did not induce an inflammatory response in any tissue, nor did it cause any noticeable adverse effects on hematological parameters. Furthermore, formulations at low paramagnetic ion concentrations enable significant and sustained contrast enhancement at high magnetic fields [61]. Chawda et al. [62] engineered a multifunctional nanovehicle for multimodal bioimaging and drug delivery. Starting from gadolinium-decorated reduced graphene oxide nanosheets (rGO-Gd-DTPA), a high T_1_ NMR efficiency was achieved (r_1_ = 16.8 s^−1^ mM^−1^ at 1.5 T) and the optical responsive bare GO for swept-source optical coherence tomography was targeted. In addition, drug release was estimated at approx. 92% within 3 days. 

## 4. Conclusions

Over the past years, carbon-based materials designed as highly efficient MRI contrast agents have made substantial progress toward reliable clinical applications. Combining paramagnetic species such as gadolinium or manganese (either as ions or as chelates) with carbon-based nanoprobes is a straightforward strategy to produce contrast agents displaying enhanced relaxivities while providing multifunctionality, thanks to the variety of functional groups present on the nanomaterials’ surface. The major challenge associated with the use of carbon-based nanomaterials is their native insolubility in aqueous media. In this context, stabilization strategies based on the oxidation of functional groups or their functionalization with polymer chains were proven effective for increasing the dispersion in aqueous media to concentrations in the millimolar range and above. Numerous systems are particularly interesting as highly efficient contrast agents, but are, however, at the stage of the proof of concept, mainly in preclinical studies on animal models. In the near future, a major key point in carbon-based materials research will be the meticulous establishment of the interplay between the structure, pharmacokinetics, and toxicity of the developed systems to pave the way for their translation into the clinic.

## Data Availability

Data is contained within the article.
